# Evaluation of New Zealand’s High-Seas Bottom Trawl Closures Using Predictive Habitat Models and Quantitative Risk Assessment

**DOI:** 10.1371/journal.pone.0082273

**Published:** 2013-12-16

**Authors:** Andrew J. Penney, John M. Guinotte

**Affiliations:** 1 Australian Bureau of Agricultural and Resource Economics and Sciences Ministry of Fisheries, Canberra, Australian Capital Territory, Australia; 2 Marine Conservation Institute, Bellevue, Washington, United States of America; The Australian National University, Australia

## Abstract

United Nations General Assembly Resolution 61/105 on sustainable fisheries (UNGA 2007) establishes three difficult questions for participants in high-seas bottom fisheries to answer: 1) Where are vulnerable marine systems (VMEs) likely to occur?; 2) What is the likelihood of fisheries interaction with these VMEs?; and 3) What might qualify as adequate conservation and management measures to prevent significant adverse impacts? This paper develops an approach to answering these questions for bottom trawling activities in the Convention Area of the South Pacific Regional Fisheries Management Organisation (SPRFMO) within a quantitative risk assessment and cost : benefit analysis framework. The predicted distribution of deep-sea corals from habitat suitability models is used to answer the first question. Distribution of historical bottom trawl effort is used to answer the second, with estimates of seabed areas swept by bottom trawlers being used to develop discounting factors for reduced biodiversity in previously fished areas. These are used in a quantitative ecological risk assessment approach to guide spatial protection planning to address the third question. The coral VME likelihood (average, discounted, predicted coral habitat suitability) of existing spatial closures implemented by New Zealand within the SPRFMO area is evaluated. Historical catch is used as a measure of cost to industry in a cost : benefit analysis of alternative spatial closure scenarios. Results indicate that current closures within the New Zealand SPRFMO area bottom trawl footprint are suboptimal for protection of VMEs. Examples of alternative trawl closure scenarios are provided to illustrate how the approach could be used to optimise protection of VMEs under chosen management objectives, balancing protection of VMEs against economic loss to commercial fishers from closure of historically fished areas.

## Introduction

United Nations General Assembly Resolution 61/105 on sustainable fisheries [1] calls upon regional fisheries management organisations to establish measures requiring participants in bottom fisheries to assess, on the basis of the best available scientific information, whether fishing activities would have significant adverse impacts on vulnerable marine ecosystems (VMEs), and to close areas where VMEs are known or are likely to occur, unless conservation and management measures have been established to prevent significant adverse impacts on those VMEs. These requirements were incorporated into interim measures for bottom fisheries adopted by participants in the negotiations to establish the South Pacific Regional Fisheries Management Organisation [2].

The FAO International Guidelines for the Management of Deep Sea Fisheries in the High Seas [3] include advice on broad characteristics of VMEs and guidelines on what might constitute a significant adverse impact. However, these guidelines provide no advice on what might constitute adequate measures to prevent significant adverse impacts. In 2009, the UN General Assembly reaffirmed resolution 61/105 and emphasized the need for full implementation in UNGA Resolution 64/72 [4]. Three difficult questions arise from these UNGA Resolutions for managers charged with conducting risk assessments and implementing measures to prevent significant adverse impacts:

1. What are vulnerable marine ecosystems and where are these likely to occur?
2. What constitutes a significant adverse impact and how can the likelihood of interaction and risk of fisheries impact on VMEs be assessed?
3. What might qualify as adequate conservation and management measures to prevent significant adverse impacts?

This paper describes an approach for addressing these questions for bottom trawling activities in the SPRFMO Convention Area. The first two questions are addressed using a quantitative risk assessment framework [5], [6] using catch and effort data for the New Zealand high-seas bottom trawl fishery and predicted model results for deep sea coral (scleractinian) habitat suitability [7]. Optimisation of spatial protection planning options to address the third question is explored using cost : benefit analysis to evaluate spatial closures implemented by New Zealand for eight high seas fishing areas under the interim SPRFMO bottom fishing regulations [8], [9] and to compare these with alternative closure scenarios.

**Figure 1:**
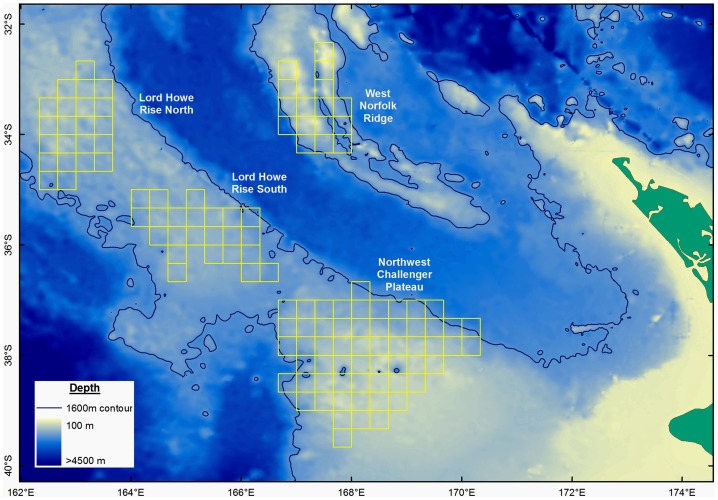
Characteristics of the high-seas bottom trawling areas in the Tasman Sea west, of New Zealand. Shaded bathymetric depth of the Challenger Plateau, Lord Howe Rise and West Norfolk Ridge fishing areas to the west of New Zealand, showing the 1600‘fishable depth’ areas. Yellow 20-minute latitude/longitude blocks show the New Zealand bottom trawling footprint fished by New Zealand vessels in this western portion of the study area over the years 2002 to 2006. New Zealand has similarly mapped the bottom trawl footprint along the Louisville Ridge in 20-minute blocks.

An overview of the seabed topographic characteristics of the northern Tasman Sea study area west of New Zealand is shown in Figure 1. Shaded bathymetry shows the extensive plateaus and ridges constituting the important Challenger Plateau, Lord Howe Rise and West Norfolk Ridge areas fished by New Zealand bottom trawlers. The high seas portions of these fishing areas fall under the management jurisdiction of the South Pacific Regional Fisheries Management Organisation (SPRFMO), whose Convention entered into force in August 2012. Under interim conservation and management measures adopted in 2007 by participants in the negotiations to establish SPRFMO, participants in bottom fisheries in the SPRFMO Convention Area are required to limit bottom fishing activities to within areas fished over the period 2002–2006 [2], with these fished areas being mapped as a ‘footprint’ of fished 20-minute latitude/longitude blocks. The 20-minute blocks constituting the New Zealand bottom trawl footprint on the Challenger Plateau, Lord Howe Rise and West Norfolk Ridge over the period 2002–2006 are shown in Figure 1. New Zealand has similarly mapped their trawl fishing footprint as 20-minute blocks along the Louisville Ridge, east of New Zealand. Within this bottom trawl footprint, New Zealand has established spatial closures to protect vulnerable marine ecosystems by closing 40% of the blocks constituting the total New Zealand bottom trawl footprint.

## Methods

### Catch and Effort Data

Catch and effort data for New Zealand high-seas bottom trawling in the SPRFMO Area were obtained from the New Zealand Ministry of Fisheries commercial catch and effort database. 1990 is the first full year represented in this database and 2006 is the end of the 2002–2006 reference period chosen by SPRFMO participants as the basis for mapping historically fished areas. 2002–2006 was also the time period covered by the data analyses used to develop the New Zealand management measures for their SPRFMO Area bottom fisheries [9].

This database includes data for foreign-flagged vessels that operated under charter to New Zealand companies. Whereas foreign flag charter vessel data were excluded from the SPRFMO Area impact assessment developed by New Zealand [8], [9], data for all vessels have been included in this paper to ensure comprehensive mapping of fishing effort for impact assessment purposes. New Zealand vessels have conducted about 90% of the fishing effort in this region, with little evidence of illegal, unreported or unregulated (IUU) fishing. Inclusion of data from 1990–2001, and for foreign charter vessels, extended the bottom trawled area outside the 2002–2006 footprint published by the New Zealand Ministry of Fisheries (Figure 1) [8]. For the purposes of evaluating the spatial closures implemented by New Zealand within the bottom trawl footprint in these fishing areas, catch and effort analyses were restricted to the tows that were conducted within the 20-minute blocks constituting the New Zealand SPRFMO Area bottom trawl footprint over the period 2002–2006 [9].

Bottom trawl data for the period 1990–2006 were retrieved from the high seas versions of the Trawl Catch Effort and Landings Return (TCELR) forms, which provide tow-by-tow information with start and end date, time and location, fishing method, depth and estimated catch by species (kg) for each tow. This is primarily an orange-roughy (*Hoplostethus atlanticus*) targeted fishery and data were error-checked using standardised procedures routinely used for orange roughy-targeted trawl catch and effort analyses for this fishery [10], [11]. Error checks were performed for fishing position, depth, tow speed, duration, distance and target species [12]. Additional comprehensive geospatial (tow start and end position) error checking and correction was conducted using procedures described in Penney [13]. Records were excluded for tows with no fishing position information or which fell within Exclusive Economic Zones (EEZs). Two minor fishing areas near the Kermadec Islands and New Caledonia had only one trawl per 20-minute footprint block, contributed negligible catches, and were excluded from the analyses.

### Mapping of Historical Bottom Trawl Effort

New Zealand fishers reported 43,289 bottom trawl tows in the SPRFMO Area over the period 1990–2006. Of these, 39,902 tows had reliable position information, 1,627 appeared to have east/west errors, and 1,760 clearly had unreliable positions. Original data forms, observer reported tow positions and vessel monitoring system data were checked for all of the erroneous positions, resulting in the correction of 1,716 tows, including most of the tows with east/west position errors. The remaining 1,671 (4%) erroneous tows were excluded from analyses.

All valid trawl tows for the period 1990–2006 were imported into ArcGIS©, incorporating a randomised jitter up to 0.5 minutes either side (latitude and longitude) of the reported positions to compensate for rounding to the nearest minute of reported start and end positions [13]. Tows were geospatially cropped to the 1600 m depth contour, or to fishing effort bounding polygons [13] in areas where GEBCO data [14] appeared to be inadequate. This provided an analysis dataset of 41,618 high-seas bottom trawl tows occurring within fishable depths over the period 1990–2006, including reported orange roughy and total top ten species catches per tow. The depth of 1500 m has previously been reported as the maximum depth fished by New Zealand bottom trawlers on the high seas [10], [11]. The maximum fishing depth has been extended slightly in this analysis to 1600 m, based on geospatial analysis of the depth range of trawl tows and comparison with GEBCO bathymetric data.

Tow lengths were determined in ArcGIS© using an Albers equal area conic projection (which provides proportionally correct area estimates) and tow lines were then split by the boundaries of the 20-minute blocks constituting the New Zealand 2002–2006 SPRFMO Area bottom trawl footprint. Although the Albers projection does not conserve length, there is negligible distortion of length across the width of a 20-minute blocks (∼32 km), and these lengths were only used to determine proportional catches per segment within each block. The lengths of split tow segments within each block were determined and the proportional orange roughy and top ten species catches for each tow segment were calculated from the ratio of the tow segment length over the full tow length. The resulting tow segment data were summed by footprint block to determine the total number of tows (segments), the summed (cumulative) tow length and the total reported orange roughy and top ten species catches within each footprint block over the period 1990–2006.

### Estimation of Seabed Swept Areas

Two alternative measures of seabed swept are used in this paper, for two different purposes. Cumulative swept area is the simple sum of estimated areas swept over time, with individual areas of all tows simply being added together, without accounting for any overlap in tows. Cumulative swept area provides a measure of the repetitive impact in an area over time. This is essentially a measure of fishing intensity and is an appropriate measure of the increasing likelihood of interaction with vulnerable marine ecosystems in repetitively trawled areas. However, this measure ignores the fact than many tows may overlap and does not measure the area of the seabed that was actually swept. Actual swept area was therefore estimated by first merging overlapping trawls and then estimating the swept area of the resulting merged tows. This provides a measure of the seabed area that has actually been swept, correcting for any overlap in tows. This is an appropriate measure of the area of the seabed that has actually been impacted by fishing operations, for use in discounting the biodiversity or habitat suitability of an area.

New Zealand vessels fishing in the SPRFMO Area average 48 m in length [15] and Baird et al. [16] applied a swept width between trawl doors in the orange-roughy fishery of 100 m for vessels up to 46 m length (S. Baird, NIWA, pers comm). The summed lengths of tow segments within each footprint block were therefore converted to estimates of cumulative swept area per block by multiplying the cumulative tow length by an assumed swept width of 0.1 km (100 m) between trawl doors.

Even after jittering of tow start and end positions, many tow lines overlap, particularly in heavily fished areas. Actual swept area within each trawl footprint block was estimated using ArcGIS© to generate polygon buffers 50 m either side of each of the tow lines (assuming 100 m door spread width). These buffered tow lines were dissolved into merged swept-area polygons which were then split by the boundaries of the trawl footprint blocks. Actual swept areas over the period 1990–2006 were calculated as the sum of the areas of the dissolved, split, buffered tow polygons within each block.

The planar surface area of 20-minute bottom trawl footprint blocks decreases polewards as a result of convergence of longitudinal meridians. The New Zealand bottom trawl footprint blocks average 1,088 km^2^ in area, decreasing from 1,240 km^2^ in the Fiji Basin to 901 km^2^ at the southern end of the Louisville Ridge (Albers equal area conic projection). Any particular trawl swept area will therefore impact a greater proportion of the area of a footprint block towards the south of the fished regions than towards the north. To enable comparison between blocks, cumulative and actual swept areas within each block were expressed as proportions of the total area of the blocks within which they occurred.

To enable comparison of seabed swept areas with areas of available fishable depth, the proportion of fishable depth within each footprint block was determined from the proportion of data points in the GEBCO 30 arc-second (∼1 km^2^) bathymetric data set [14] that are <1600 m depth within each block. Cumulative and actual swept areas within each block were expressed as proportions of the planar area of fishable depth in the blocks within which they occurred, to provide indices of the cumulative and actual swept proportion of the fishable area in each block.

### Predictive Habitat Models

**Figure 2:**
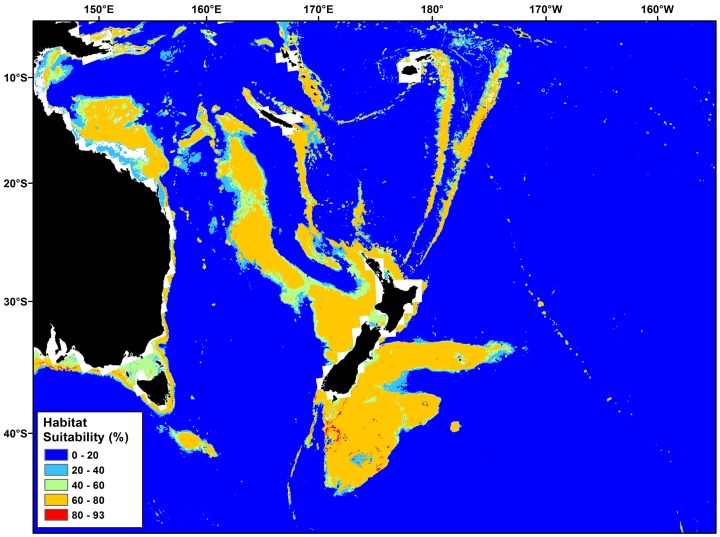
Predicted scleractinian coral habitat suitability (*Goniocorella dumosa, Solenosmilia variabilis, Madrepora oculata, Enallopsammia rostrata* and *Oculina varicose)* in the New Zealand region (Davies &Guinotte 2011).

The global deep-sea scleractinian coral habitat suitability model developed by Davies & Guinotte [7] was used to generate indices of the likelihood of occurrence of VMEs within each of the New Zealand high seas bottom trawl footprint blocks. This is a 30 arc-second (∼1 km^2^) resolution maximum entropy (Maxent) model that predicts habitat suitability for six species of deep-sea, habitat-forming scleractinian corals (*Enallopsammia rostrata*, *Goniocorella dumosa*, *Lophelia pertusa*, *Madrepora oculata*, *Oculina varicosa* and *Solenosmilia variabilis*), using global databases for 15 bathymetric, hydrographic, chemical and biological predictor variables. This global model incorporated all the available scleractinian reef-forming coral occurrence records for thespecies occurring in the New Zealand region from Tracey *et al*. [35] (n = 631; *G. dumosa* = 204; *S. variabilis* = 191; *M. oculata* = 118; *E. rostrata* = 98; *O. varicosa* = 20; *L. pertusa* does not occur in the New Zealand region). The predicted, combined habitat suitability for these species in the New Zealand region is shown in Figure 2 from Davies & Guinotte [7].

This scleractinian habitat model was used to generate 1600 data points (about one per 1 km^2^) for each of the 20-minute blocks in the New Zealand SPRFMO Area bottom trawl footprint. Each data point included position (latitude/longitude), depth (from the underlying 30-arc-sec bathymetric data), and the overall predicted habitat suitability (0–100%) for all scleractinian species combined. The scleractinian habitat suitability values for data points within each block were averaged over all depths to provide indices of overall coral habitat suitability per block, and over fishable depths (0–1600 m) to provide indices of fishable-depth coral habitat suitability per block.

### Quantitative Risk Assessment

The multi-level approach to Ecological Risk Assessment for Effects of Fishing (ERAEF) developed by Hobday *et al*. [5], [6] has become internationally well established. In particular, the intermediate (level-2) quantitative risk assessment approach using multi-component, scored productivity/susceptibility analysis (PSA) plots has been widely adopted as a standard approach to generating two-dimensional, integrated measures of risk for fishery resources [6], [17], [18]. Although originally developed for evaluating risks to fish stocks, ERAEF has more recently been adapted to evaluate the risk of benthic impacts of fishing [19].

The ERAEF productivity–susceptibility analysis approach was further adapted in this study to directly address the two main questions arising out of y UNGA resolution 61/105: where are VMEs likely to occur? and what is the risk of fisheries interaction with these VMEs? These questions were expressed as the axes of a two-dimensional analysis, similar in concept to the PSA plots used in level-2 ERAEF assessments. Likelihood of VME Occurrence was plotted against Likelihood of Fishery Interaction to quantify the risk of significant impacts on VMEs in each footprint block. Risk indices for each of the 20-minute trawl footprint blocks along these two axes were quantified as described below.

#### Likelihood of VME occurrence

The ERAEF PSA approach uses quantified or ranked answers to a range of questions to provide integrated measures of productivity based on a number of measures or indicators [5], [6]. Similarly, predictive habitat models predict the likelihood of favourable habitat for VMEs, in this case deep-sea corals, using a wide variety of predictor variables. These models therefore provide multi-factorial, integrated measures of the likelihood of favourable habitat that can be directly used as indices of likelihood of occurrence of the VMEs concerned.

The Scleractinia-combined habitat suitability values from Davies & Guinotte [7] were used as indices of the VME-Likelihood (x-axis) values per footprint block. Although technological advances may extend the trawlable depth range in future, the risk at depths greater than 1600 m is currently zero for this fishery. Risk assessments for effects of fishing can therefore be confined to the fishable depth portion of each footprint block. For the purposes of risk assessment, the average habitat suitability values per footprint block for the VME-Likelihood axis were calculated using only the fishable depth (0–1600 m) habitat suitability points within each block.

Predicted fishable-depth VME likelihood values were then discounted for the effects of historical fishing in each block. The impacts of trawling, particularly the removal of fragile, habitat-forming species and resulting reduction in biodiversity, have been well documented [20], [21], [22], [23], [24], [25]. There is evidence that recovery of these impacted deepwater areas is extremely slow. Waller *et al*. [26] and Rogers *et al*. [27] report total denudation of trawled areas on the Corner Rise seamount complex in the northwest Atlantic, with little sign of recovery after periods of 20 to 40 years. Williams *et al*. [28] found no evidence of recovery in multivariate assemblage patterns for historically trawled areas on New Zealand and Australian seamounts over a 5–10 year timeframe following cessation of trawling in those areas. Recent work on age determination of the dominant New Zealand region habitat forming scleractinian coral *Solenosmilia variabilis* by Neil *et al*. [29] have indicated that re-establishment of small colonies could take hundreds of years, while re-establishment of large colonies (2–3 m across) could take thousands of years. The degree to which seabed biodiversity is likely to have been reduced in fished areas is therefore an important factor to consider in risk assessments and when evaluating the cost-benefit of alternative spatial closures.

Residual biodiversity discounting factors should ideally be determined from properly designed control-impact seabed biodiversity surveys in fished and unfished areas. However, no such surveys have been conducted for any of the SPRFMO bottom-trawled areas. Noting observations by Koslow *et al*. [22], [23] and Waller *et al*. [26] regarding denudation of bottom trawled areas, for the purpose of determining discounting factors in this paper, it was assumed that residual predicted habitat suitability in actual swept areas was zero. Assuming that coral occurrence in swept areas has been reduced to zero results in discounted overall habitat suitability values per block being inversely proportional to the proportion of the fishable depth area that has actually been swept. For example, if half of the fishable depth area has been swept, then the discounted habitat suitability index for the fishable depth area will be half of the original average habitat suitability for the fishable depth area. Resulting discounted, fishable depth, habitat suitability values for each footprint block were used for the VME-Likelihood axis in risk assessment plots.

#### Likelihood of fishery interaction

The y-axis on ERAEF productivity-susceptibility plots measures the susceptibility of fisheries or areas to a particular impact. In the context of the questions posed by the UNGA requirements, the comparable y-axis in the risk assessments presented here measures the likelihood of fisheries interaction with VMEs in each of the footprint blocks. Of the two measures of seabed impact calculated, cumulative swept area (being a measure of fishing intensity) provides the most appropriate indicator of the likelihood of fishery interaction with VMEs. Areas that are repeatedly trawled each year are of more interest to the fishery and will have a higher likelihood of ongoing fisheries interaction with any residual VMEs. Cumulative swept area values for each footprint block were therefore used for the Fishery-Interaction axis in risk assessment plots.

### Cost : Benefit Analysis of Alternative Spatial Closures

The predicted, discounted likelihood of occurrence of VMEs provides a measure of the potential benefit of closing each trawl footprint block, in terms of meeting UNGA requirements to protect areas likely to contain VMEs. Provided some meaningful measure of cost to industry of the closure of alternative footprint blocks can be calculated, the cost : benefit trade-off of alternative spatial closure scenarios can be evaluated and optimised against any specified cost and benefit objectives.

For analysis of previously fished areas, quantitative measures of historical catch and effort can provide indices of fishing industry interest in an area. Strictly, these are retrospective measures of the cost that would have been incurred if those blocks had been closed historically. However, if the intention is to maintain stocks at sustainable levels in each area, rather than to pursue a policy of sequential depletion and movement to new fishing areas, then historical catch and effort provide appropriate measures of the ongoing suitability of the area for the fish species concerned, and of potential future value of the area to the fishing industry.

New Zealand’s high-seas spatial closures currently involve the closure of entire 20-minute footprint blocks, irrespective of depth [9]. However, closure of a block with a small area of fishable depth, and therefore with little area of high suitability for stony corals, and at no risk from fishing, will be of less benefit than closing a block lying entirely within fishable depth. In contrast to the approach taken in risk assessments, for the purposes of cost : benefit analysis of existing closures, average habitat suitability should be determined across the entire depth range of each block, and not just across fishable depth range. The average, all-depths VME likelihood of each trawl footprint block was therefore calculated using habitat suitability values for all points in each block across all depths, and not just fishable depth, after discounting the habitat suitability of points within the actual fishable depth swept areas in each block to zero to account for reductions in biodiversity as a result of trawling.

Either effort or catch could be used as measures of industry interest in particular areas, and therefore of the cost of closing those areas. However, loss of catch provides a more direct measure of cost of closures to industry than effort. The cost to industry of closing particular footprint blocks was therefore calculated as the total historical catch of the top ten species (Table 1) within each block over the period 1990–2006. Although orange roughy dominate catches, alfonsino (*Beryx splendens, B. decadactylus*) and/or oreos (*Allocyttus*, *Pseudocyttus* and *Neocyttus* species) have contributed substantial catches in some areas or years. The top ten species catch was therefore considered to be a better measure of cost than orange roughy alone. While the relevance of historical catch as a measure of cost to industry may be questioned, it is worth noting that, during a marine protected area (MPA) planning process for the Antarctic Ross Sea region [30], the fishing industry themselves chose historical fishing effort as their preferred measure of the cost to industry of alternative MPA proposals.

**Table 1:** Total reported all-areas bottom trawl catch (t) of the top ten species/groups, and of all species, by New Zealand flagged and foreign flag charter vessels in the convention area of the South Pacific Regional Fisheries Management Organisation (SPRFMO) over the period 1990–2006.

Common Name	Latin Name	1990–2006 Catch (t)		
		NZ Flag	Other Flag	All Flags
Orange roughy	*Hoplostethus atlanticus*	49,515	11,374	60,889
Black cardinalfish	*Epigonus telescopus*	3,875	206	4,081
Black oreo	*Allocyttus niger*	1,748	399	2,146
Smooth oreo	*Pseudocyttus maculatus*	1,428	140	1,567
Alfonsino	*Beryx splendens/B. decadactylus*	1,049	211	1,260
Ribaldo	*Mora moro*	345	111	456
Spiky oreo	*Neocyttus rhomboidalis*	371	55	426
Rattails	Macrouridae	320	13	334
Seal shark	*Dalatias licha*	165	7	172
Boarfish	*Pseudopentaceros richardsoni, Paristiopterus labiosus*	124		124
Total top ten species catch		58,940	12,515	71,455
Total all species catch		60,899	16,451	77,350

–2006 bottom trawl footprint prior to 2002, and so are higher than the fishing area totals in Table 2. These include catches made outside the New Zealand 2002

#### Evaluation of alternative closure scenarios

Existing spatial closures in the New Zealand SPRFMO bottom trawl footprint close 40% of the blocks across the entire footprint [9]. However, existing closures do not close 40% of the blocks within each fishing area, with some fishing areas having more, and some areas having less, than 40% of blocks closed. The decision to close 40% of blocks across the footprint was a choice by managers within the 30% to 50% range for representative closures recommended by Clark [31], Lauck *et al*. [32], Botsford *et al*. [33], Airame *et al*. [34] and Rogers *et al*. [27]. For the purposes of evaluating alternative spatial closure scenarios in this paper, it was assumed that closure of 40% of the footprint blocks remained a management objective. However, in order to ensure regional representation of closures, it was further assumed that the objective should be to close 40% of the blocks within each of the fishing areas, and not just across the footprint, to ensure representivity by fishing area. This approach taken in this analysis therefore differs from that used by New Zealand, and this has consequences for the optimisation of spatial closures (see cost : benefit analysis results).

Having determined the number of blocks (40%) to be closed in each fishing area, cost : benefit trade-off curves for alternative spatial closure scenarios were generated. The starting closure scenario for each fishing area was generated by sorting the footprint blocks in descending order of discounted, all-depths, average habitat suitability and closing the 40% of blocks with highest average habitat suitability. This starting scenario provides the highest overall, average, discounted habitat suitability that can be achieved by any 40% closure of blocks within each fishing area. Scenarios of decreasing overall average habitat suitability were then generated by opening the closed block with the highest historical catch (cost) and closing the block with the next highest average habitat suitability. This process was repeated, re-calculating the average habitat suitability and total cost of the revised closures at each step, until all blocks in each fishing area had been accounted for.

This process generates cost : benefit trade-off curves starting from the closure scenario of highest average habitat suitability (benefit), and ending with the closure scenario of least historical catch lost (cost), with each sequential scenario along these curves having decreasing cost to industry, as well as decreasing average coral habitat suitability. To provide visually reciprocal declining conservation benefit and increasing retained catch curves, the cost to industry was plotted as percentage retained catch. The benefit and cost of the existing closures in each fishing area were calculated in the same way and plotted as points on these optimisation curves to provide a direct comparison of the value and cost of existing closures with the explored range of alternative scenarios.

#### Optimisation of spatial closures

Cost : benefit trade-off curves for each fishing area can be used to select an ‘optimal’ spatial closures at some point along the trade-off, given specified management objectives in terms of benefit and cost. Selection of a preferred position along the trade-off curves would usually be based on an iterative consultation between fisheries managers, industry representatives and other stakeholders, using pre-agreed objectives for each axis, comparing conservation benefit of spatial closures *vs*. cost to industry resulting from lost access to fishing areas. Such a process is described by Sharp and Watters [30] for the Ross Sea MPA planning process. The objectives to be pursued would also typically be established by managers in consultation with stakeholders. However, for the purpose of generating illustrative ‘optimised’ closure examples in this paper, the following example management objectives were assumed:

1. To achieve protection of at least 75% of the achievable range in average habitat suitability (maximum to minimum) across the alternative scenarios in each fishing area (75% of the benefit axis range).
2. To retain at least 75% of the historical top ten species catch in each fishing area (75% of the retained catch axis).

An optimisation approach such as this involves an explicit balancing of competing objectives related to maximising conservation and catch, using objective and quantitative measures for each axis. In generating examples of ‘optimised’ closure scenarios against these management objectives, the conservation objective was initially given precedence. If the fisheries cost reduction objective could not be achieved while retaining 75% of potential habitat suitability, then retained catch was allowed to decrease below 75% of historical catches to retain at least 75% of potential habitat suitability. However, if both objectives could be achieved across a range of alternative closure scenarios, then within this range of ‘acceptable’ alternatives, the objective of reducing cost to industry was given precedence, so that retained catch was maximised after ensuring protection of at least 75% of potential habitat suitability.

## Results

### New Zealand’s Historical High Seas Bottom Fishing Catch

New Zealand flagged bottom trawl vessels and foreign charter vessels operating for New Zealand companies reported a total high-seas bottom trawl catch of 77,350 t of all species in the SPRFMO Area over the period 1990–2006 (Table 1). This includes catches made outside the New Zealand 2002–2006 SPRFMO Area bottom trawl footprint prior to 2002 [9]. The top ten species contributed 92% of this catch, with orange roughy contributing 79% of the all species catch and 85% of the top ten species catch. The other top ten species, black cardinalfish (*Epigonus telescopus*), oreos (black oreo *Allocyttus niger*, smooth oreo (*Pseudocyttus maculatus)*, spiky oreo (*Neocyttus rhomboidalis*), alfonsino, ribaldo (*Mora moro*), rattails (Macrouridae), seal shark (*Dalatias licha*) and boarfish (*Pseudopentaceros richardsoni, Paristiopterus labiosus*), together contributed 12% of the total reported catch (Table 1).

### Distribution of Historical Bottom Trawl Effort and Impact by Fishing Area

The fishable depth areas of the eight fishing areas constituting the New Zealand SPRFMO Area 2002–2006 bottom trawl footprint, and the total amount of fishing effort in each fishing area over the period 1990–2006, are summarised in Table 2. The western fishing region (Lord Howe Rise, Challenger Plateau, West Norfolk Ridge and Three Kings Ridge) is almost three times the area of the eastern (Louisville Ridge) region and has the longest fishing history, particularly the NW Challenger Plateau, where high-seas bottom trawling started in the late 1980 s as an extension of the inside-EEZ fishery. The differences in historical fishing effort are partially attributable to differences in seabed topography between the western and eastern regions. The western region consists of easily accessible, large plateau and ridge features that lie predominantly within fishable depth, whereas fishable areas along the Louisville Ridge are confined to the summits of distant, relatively small, discrete seamounts. Over 90% of the bottom trawl footprint in the western region lies within fishable depth (0–1600 m) whereas only 14% of the Louisville Ridge footprint lies at fishable depths (Table 2).

**Table 2:** Number of 20-minute latitude/longitude bottom trawl footprint blocks, total footprint area (km^2^), fishable depth (0 m–1600 m) area (km^2^), cumulative tow length (km), cumulative and actual swept areas (km^2^) and reported catches (t) by New Zealand flagged and foreign flag charter vessels within each of the fishing areas constituting the New Zealand bottom trawl footprint in the convention area of the South Pacific Regional Fisheries Management Organisation (SPRFMO) over the period 1990–2006.

Fishing	Number of	Total	Fishable	Total Tow	Cumulative Swept	Actual Swept	Orange Roughy	Top Ten
Area	Blocks	Area (km^2^)	Area (km^2^)	Length (km)	Area (km^2^)	Area (km^2^)	Catch (t)	Catch (t)
Lord Howe North	22	25,075	25,051	4,307	431	273	99	1,091
Lord Howe South	23	25,633	25,417	23,832	2,383	1,162	3,998	5,719
Challenger Plateau	58	62,833	59,642	179,275	17,928	8,608	12,382	16,020
West Norfolk Ridge	16	18,317	14,910	3,513	351	219	1,737	1,776
Three Kings Ridge	8	9,584	3,684	678	68	49		84
Louisville North	17	18,515	3,849	15,638	1,564	682	8,542	8,616
Louisville Central	21	21,374	2,449	30,597	3,060	933	21,394	22,008
Louisville South	12	11,456	1,071	5,602	560	213	5,341	7,624

© using an Albers equal area conic projection. All areas were calculated in ArcGIS

Total fishing effort (total tow length and cumulative swept area) in the western region is four times that on the Louisville Ridge and actual swept area in the western region is five times that on the Louisville Ridge (Table 2). The ratio between cumulative and actual swept area differs substantially between blocks depending on the degree of overlap of tows in repetitively trawled areas. In the more lightly fished West Norfolk Ridge and Three Kings Ridge fishing areas, with less overlap of tows, cumulative swept area is two to three times the actual swept area. In the more heavily fished Challenger Plateau and Louisville Ridge areas, where substantial tow overlap occurs, cumulative swept area is four to six times actual swept area over the period 1990–2006.

Across the entire western region, 7% of the all-depths footprint area, which amounts to 8% of the fishable depth footprint area, has actually been swept. Despite the fact that most of the trawl footprint on the Challenger Plateau lies within fishable depth and that this area has the longest fishing history, only 14% of the 20-minute block footprint in this area has actually been swept (Table 2). In comparison, despite lower overall fishing effort, as a result of the small area of fishable depth available on the Louisville Ridge seamounts, 25% of the available fishable depth area on the Louisville Ridge has been swept. In the more heavily fished Central Louisville Ridge area, 38% of the fishable depth area has been swept, almost three-times the 14% of fishable depth area swept on the Challenger Plateau (Table 2).

**Figure 3:**
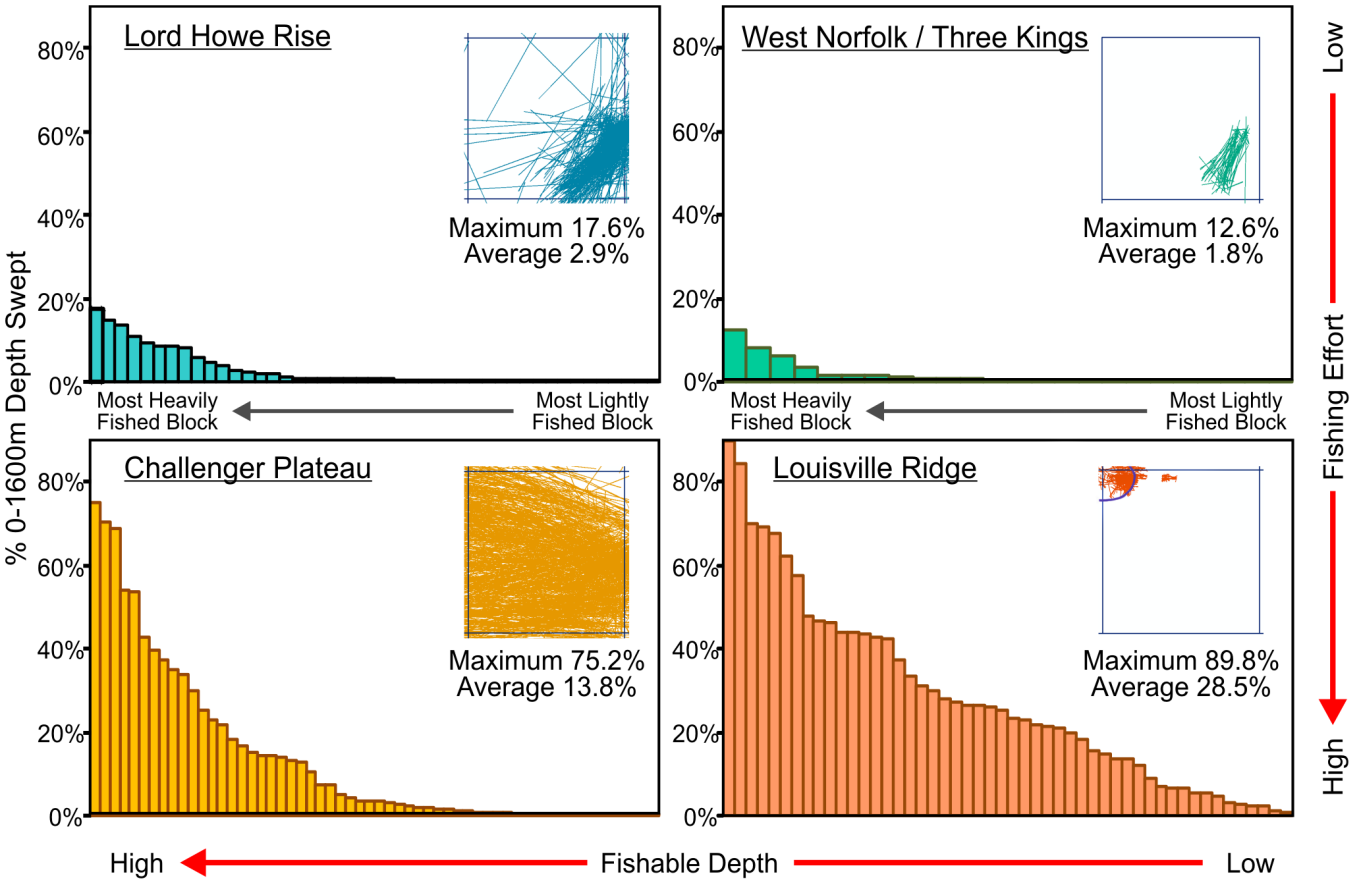
Swept area of seabed at fishable depth. Percentage of fishable depth area (0 m–1600 m) swept in each of the 20-minute blocks constituting the New Zealand SPRFMO Area bottom trawl footprint in the Lord Howe Rise (north and south combined), Challenger Plateau, West Norfolk Ridge and Three Kings Ridge (combined) and Louisville Ridge (north, central and south combined) fishing areas, sorted in descending order from most heavily to most lightly fished. Inset maps show the footprint block with the highest percentage fishable depth swept, with the maximum and average percentage fishable depth swept per block, for each fishing area. Blue contour lines show the extent of fishable depth area in the most heavily fished blocks in the Three Kings Ridge and Louisville Ridge areas.

The minimum, maximum and average percentages of the fishable depth area swept per 20-minute block in the Lord Howe Rise (north and south combined), Challenger Plateau, West Norfolk Ridge and Three Kings Ridge (combined) and Louisville Ridge (north, central and south combined) fishing areas are summarised in Table 3 and illustrated in Figure 3. The insets in Figure 3 show maps of the 20-minute footprint block with the highest proportion of fishable depth area swept in each of the fishing areas, and the maximum and average percentage fishable depth swept for each of these blocks.

**Table 3:** Summary of the estimated minimum, average and maximum percentage of the fishable depth (0 m–1600 m) area actually swept in footprint blocks in the Lord Howe Rise, West Norfolk/Three Kings Ridges, Challenger Plateau and Louisville Ridge fishing areas.

Fishing Area	% of 0 m–1600 m Area Actually Swept					Mean % 0 m–1600 m	Cumulative/
	Min	Max	Mean	StdDev	% Swept >50%	Cumulatively Swept	Actual Swept Ratio
West Norfolk/Three Kings	0.02%	12.6%	1.8%	3.1%	0%	2.8%	1.6
Lord Howe Rise	0.03%	17.6%	2.9%	4.5%	0%	5.6%	1.9
Challenger Plateau	0.01%	75.2%	13.8%	19.6%	9%	28.7%	2.1
Louisville Ridge	0.99%	89.8%	28.5%	22.6%	14%	75.5%	2.6

% of the fishable depth actually swept, the percentage of the fishable depth area cumulatively swept and the cumulative/actual swept depth ratios. These last two measures provide indices of the fishing intensity in each fishing area. Also shown are the percentages of blocks in each fishing area that were estimated to have had more than 50

The proportion of fishable depth area actually swept per block in the lightly fished Lord Howe Rise, West Norfolk Ridge and Three Kings Ridge areas averages 2% –3%, with less than 18% of the fishable area of the most heavily fished block having been swept (Table 3). In the heavily fished Challenger Plateau and Louisville Ridge areas, the percentage of fishable depth that has actually been swept averages 14% and 29% per block respectively. 75% of the fishable depth in the most heavily fished block on the Challenger Plateau has been swept and 9% of the Challenger Plateau blocks have had more than half of the fishable depth swept. 90% of the fishable depth in the most heavily fished block on the Louisville Ridge has been swept and 14% of the blocks along the Louisville Ridge have had more than half the fishable depth swept (Table 3).

### Predicted Coral Habitat Suitability in the New Zealand Region

**Figure 4:**
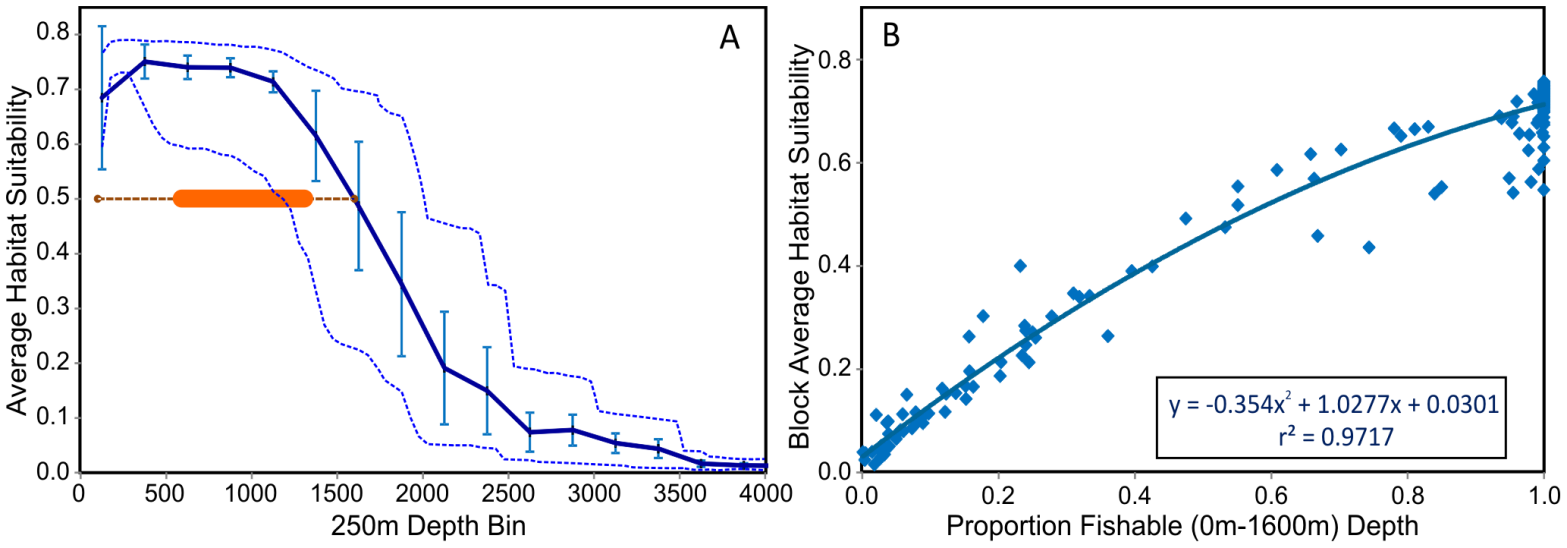
Depth – coral habitat suitability relationships. a) Predicted coral habitat suitability by depth within the New Zealand high seas bottom trawl footprint area (mean, standard deviation and range). The orange line shows the depth range (dotted line = total catch, bar = 90% of catch) over which bottom trawl catches are made; b) Relationship between the proportion of fishable depth (0 m–1600 m) and average, all-depths, predicted habitat suitability per 20-minute block in the New Zealand high-seas bottom trawl footprint. (Data from Davies &Guinotte 2011).

Predictive habitat model results from Davies & Guinotte [7] predict large areas of highly suitable habitat in the New Zealand region for a number of deepwater coral species, particularly *Goniocorella dumosa* and *Solenosmilia variabilis* (Figure 2), both of which are important habitat-forming components of deep-water benthic communities in the region [35]. There is a strong inverse relationship between depth and predicted coral habitat suitability within the New Zealand high-seas bottom trawl footprint (Figure 4a). Predicted scleractinian coral habitat suitability is high (50%–80%) across fishable depths (0 m–1600 m), decreasing rapidly below 1600 m to less than 10% below 2500 m.

The entire New Zealand high-seas bottom trawl catch has been taken in depths less than 1600 m and over 90% of the orange roughy trawl catch has been taken in 600 m–1300 m depth. Fishing effort is therefore concentrated in the depth range where predicted coral habitat suitability is highest, exceeding 60% (Fig. 2a). Using these predicted habitat model results, as a result of the strong relationship between depth and coral habitat suitability, there is a strong correlation between the proportion of fishable depth and the average coral habitat suitability in each footprint block. The average coral habitat suitability is essentially determined by the proportion of fishable depth area in each block (Figure 4b).

**Figure 5:**
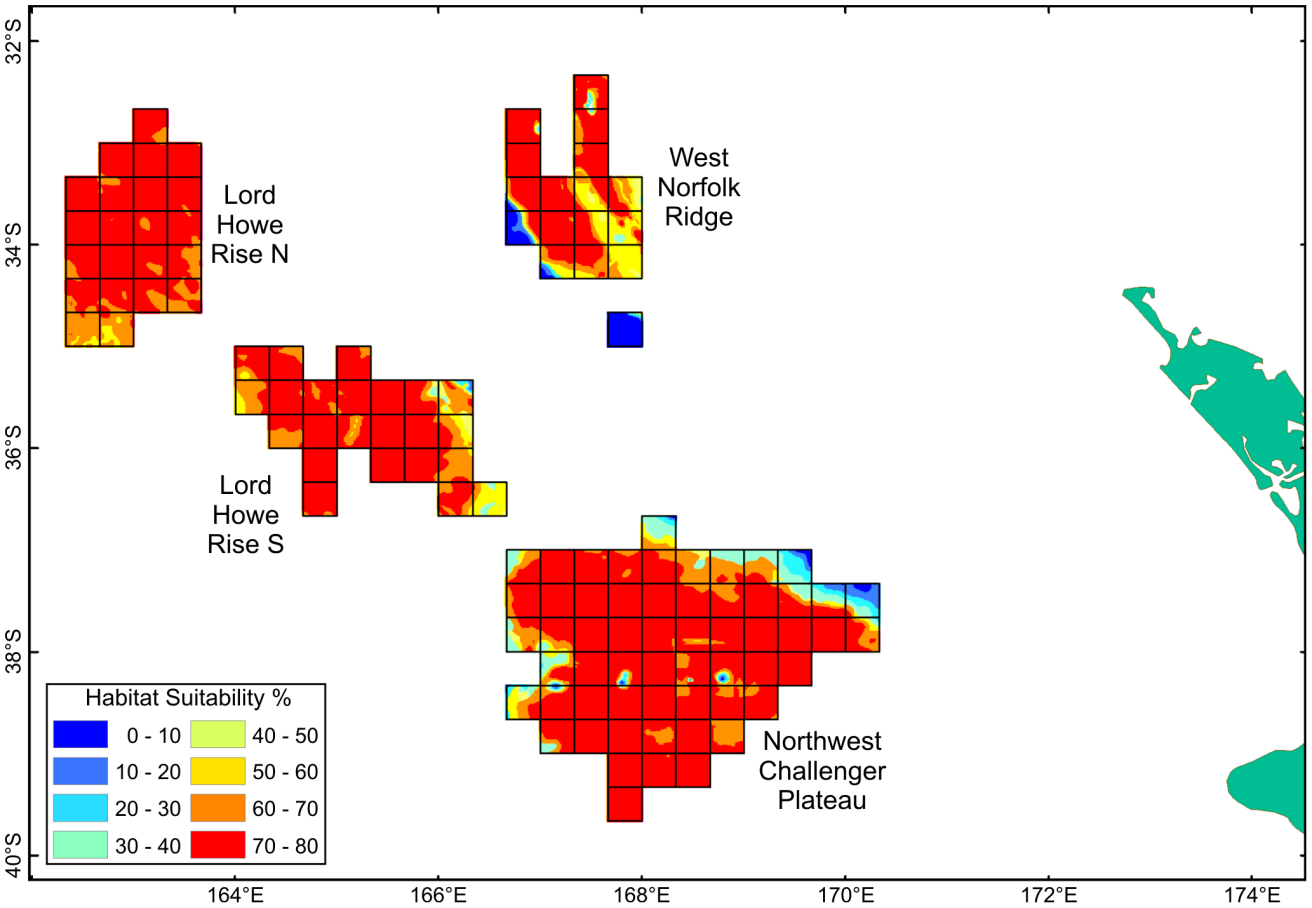
High-seas fishing footprint coral habitat suitability – western region. Distribution of predicted scleractinian coral habitat suitability in each of 20-minute latitude/longitude blocks constituting the New Zealand bottom trawl footprint in the Lord Howe Rise, Northwest Challenger Plateau and West Norfolk Ridge fishing areas (Davies &Guinotte 2011).

**Figure 6:**
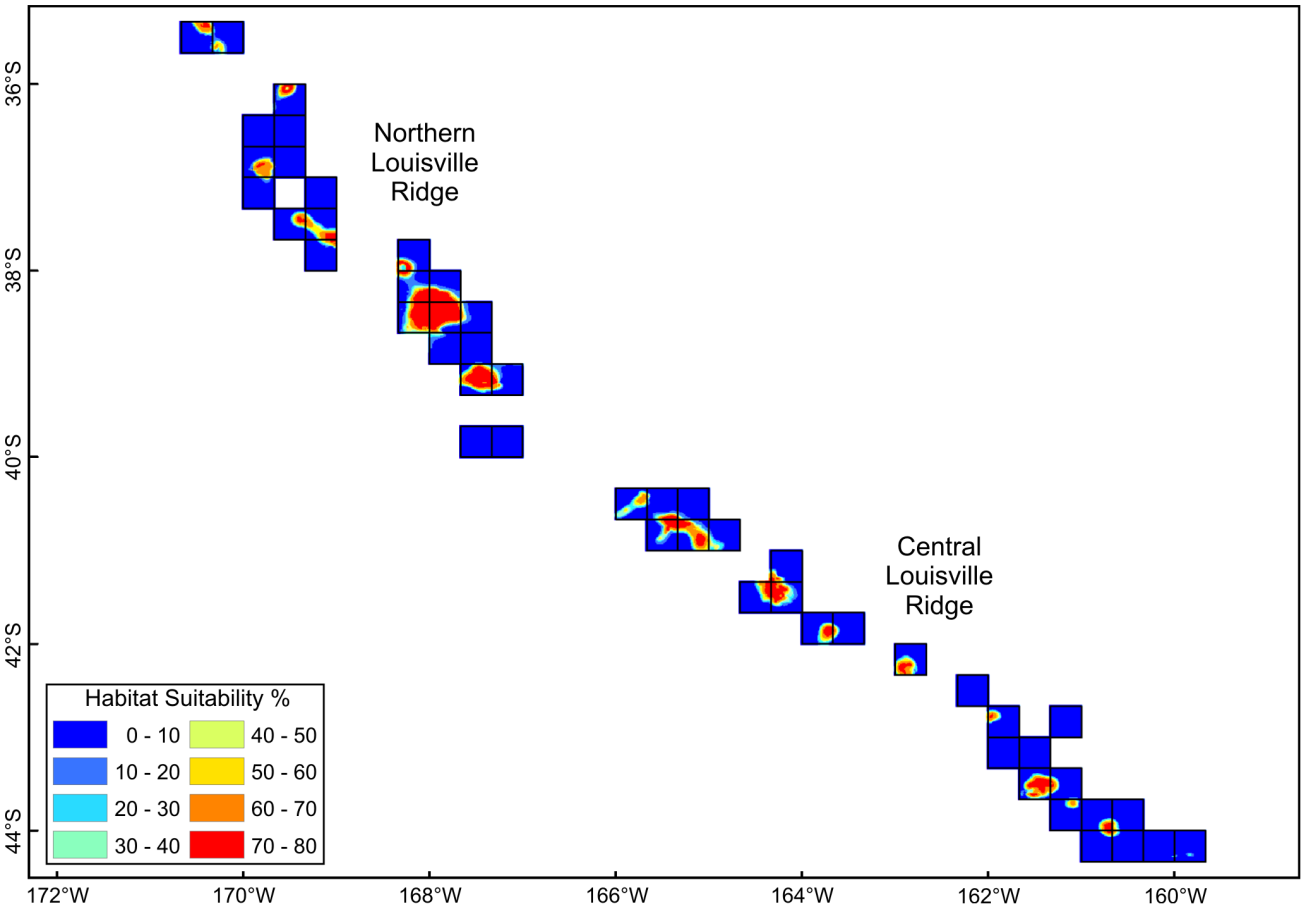
High-seas fishing footprint coral habitat suitability – eastern region. Distribution of predicted scleractinian coral habitat suitability in each of 20-minute latitude/longitude blocks constituting the New Zealand bottom trawl footprint in the Northern and Central Louisville Ridge fishing areas (Davies &Guinotte 2011).

The distribution of predicted coral habitat suitability from Davies & Guinotte [7] within each of the blocks constituting the New Zealand SPRFMO Area bottom trawl footprint is shown in Figure 5 for the Lord Howe Rise, Challenger Plateau and West Norfolk Ridge fishing areas, and in Figure 6 for the Northern and Central Louisville Ridge. As a result of the determining effect of depth on predicted coral habitat suitability, these closely resemble bathymetric charts for these areas. The high proportion of suitable coral habitat at fishable depth in the western region is particularly evident (Figure 5), as is the low proportion of suitable coral habitat, confined to seamount summits, along the Louisville Ridge (Figure 6).

### VME-Likelihood/Fishery-Interaction Risk Assessments

**Figure 7:**
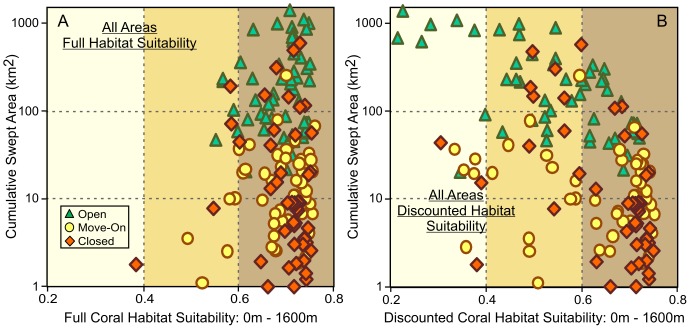
Overall VME-likelihood/fishery-interaction risk assessment analysis. Combined (all fishing areas) VME-likelihood/fishery-interaction risk assessment plots for all footprint blocks constituting the New Zealand SPRFMO Area bottom trawl footprint in all fishing areas. Coral habitat suitability is the average Davies & Guinotte (2011) Scleractinia habitat suitability values for the fishable depth (0 m–1600 m) points in each block. Risk of interaction is measured as the cumulative swept area over time in each block. Blocks have been classified by their current management status (open, move-on or closed, from Penney *et al*. 2009). a) Full fishable-depths habitat suitability, without discounting; b) Discounted fishable-depths habitat suitability, with habitat suitability of actual seabed swept areas set to zero.

Figure 7a shows the VME Likelihood - Fishery Interaction risk-assessment plot for all fishing areas combined, using the non-discounted average habitat values for fishable depths in each footprint block, plotted against the cumulative swept area per block over the period 1990–2006. The blocks have been classified according to their current management status as open, move-on, or closed [9]. The tiered distribution of block status by cumulative swept area is a direct result of open/move-on/closed status being originally determined by historical fishing effort in each block. The more heavily fished third of the blocks were left open to fishing, the more lightly fished third were closed, and the moderately fished third were made subject to a move-on rule [9]. An additional 10% of block closures in the moderately and heavily fished areas [9] is apparent as closed blocks in areas with higher cumulative swept area.

Original, non-discounted habitat suitability values per block (Figure 7a) can be used to evaluate whether the current closed blocks were comparable to the move-on and open blocks in terms of original likelihood of occurrence of VMEs before fishing. Discounted values (Figure 7b) can then be used to compare the residual VME likelihood in these block categories after discounting for the effects of fishing. In the western region (Lord Howe Rise, Challenger Plateau, West Norfolk Ridge and Three Kings Ridge), non-discounted habitat suitability values for fishable depths are virtually identical for the three block categories, averaging 69.7%, 69.7% and 70.0% for the open, move-on and closed areas respectively (Table 4). However, after discounting for the effects of fishing, the average fishable depth habitat suitability of the western region open areas is reduced to 55.4%, with only slight reduction in the average value of the move-on areas (68.7%) and closed areas (68.3%).

**Table 4:** Average, predicted habitat suitability of fishable depth areas (0 m–1600 m) in the open, move-on and closed footprint blocks in the western region (Lord Howe Rise, Challenger Plateau, West Norfolk Ridge and Three Kings Ridge) and on the Louisville Ridge (North, Central and South), showing the original, non-discounted, average habitat suitability and the remaining average habitat suitability after discounting for actual swept area in previously fished areas.

Fishing Area	Full Habitat Suitability: 0 m–1600 m			Discounted Habitat Suitability: 0 m–1600 m		
	Open	Move-On	Closed	Open	Move-On	Closed
Western Region	69.7%	69.7%	70.0%	55.4%	68.7%	68.3%
Louisville Ridge	67.7%	66.1%	63.8%	41.9%	49.3%	50.5%

Before discounting, the average fishable depth habitat suitability of open areas along the Louisville Ridge (67.7%) is similar to that of the move-on areas (66.1%) and slightly higher than that in the closed areas (63.8%) (Table 4). After discounting, average Louisville Ridge fishable depths habitat suitability values decrease to 41.9% for open areas, 49.3% for move-on areas and 50.5% for closed areas. After discounting for the effects of historical fishing, the likelihood of residual VMEs in fishable depths in closed and move-on blocks is therefore higher than in open areas in both the western and eastern regions. This indicates that, using the three effort-based management tiers, these closures are providing protection to the tier with a higher likelihood of containing VMEs.

**Figure 8:**
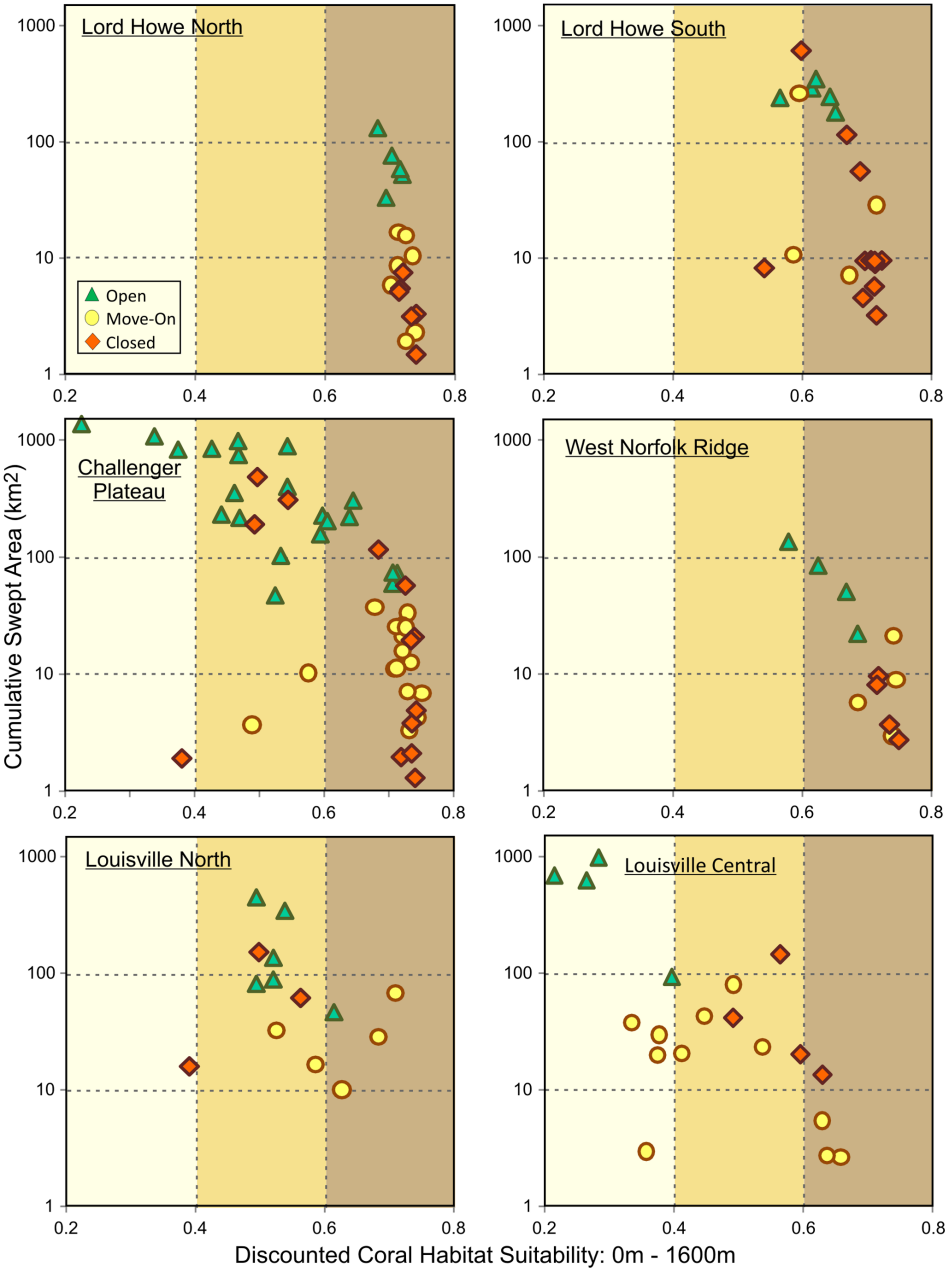
VME-likelihood/fishery-interaction risk assessment analysis by fishing area. Discounted VME-likelihood/fishery-interaction risk assessment plots by fishing area for footprint blocks in the Lord Howe Rise North and South, Challenger Plateau, West Norfolk Ridge and Louisville Ridge North and Central fishing areas. Coral habitat suitability is the average Davies & Guinotte (2011) Scleractinia-combined habitat suitability values for the fishable depth (0 m–1600 m) points in each block, discounted by setting habitat suitability of swept areas to zero. Risk of interaction is measured as the cumulative swept area over time in each block. Blocks have been classified by their current management status (open, move-on or closed, from Penney *et al*. 2009).

Individual, discounted, VME-Likelihood/Fishery-Interaction risk assessment plots for the six main fishing areas are shown in Figure 8. There is little effect of discounting in the lightly fished Lord Howe North, Lord Howe South and West Norfolk Ridge areas, where discounted fishable-depths habitat suitability remains greater than 60% for most of the blocks. Discounting results in reduction of VME habitat suitability in the more heavily fished open blocks in the Lord Howe South and West Norfolk Ridge areas. Closed blocks then have a higher average coral VME likelihood than open blocks in these two areas. The effect of discounting is greatest in the most heavily fished blocks on the Challenger Plateau, most of which are open under the current management arrangements. Due to the high proportion of fishable depth that has been swept in these blocks, discounted habitat suitability is reduced to less than 60% for most of the open blocks, while most of the closed and move-on blocks retain habitat suitability greater than 60%.

Along the Louisville Ridge, as a result of the small areas of fishable depth on seamounts and the high proportions of these areas that have been swept, discounting has a substantial effect on residual VME likelihood. On the more lightly fished Northern Louisville Ridge, the effect of discounting is moderate and the discounted VME likelihood of closed blocks remains less than that of the open or move-on blocks. However, the effect of discounting is substantial on the heavily fished Central Louisville Ridge where the residual VME likelihood of open blocks is reduced to less than 40%, with closed blocks then having a higher VME likelihood than the open or move-on blocks (Figure 8).

### Cost : Benefit Analysis of Alternative Spatial Closures

**Figure 9:**
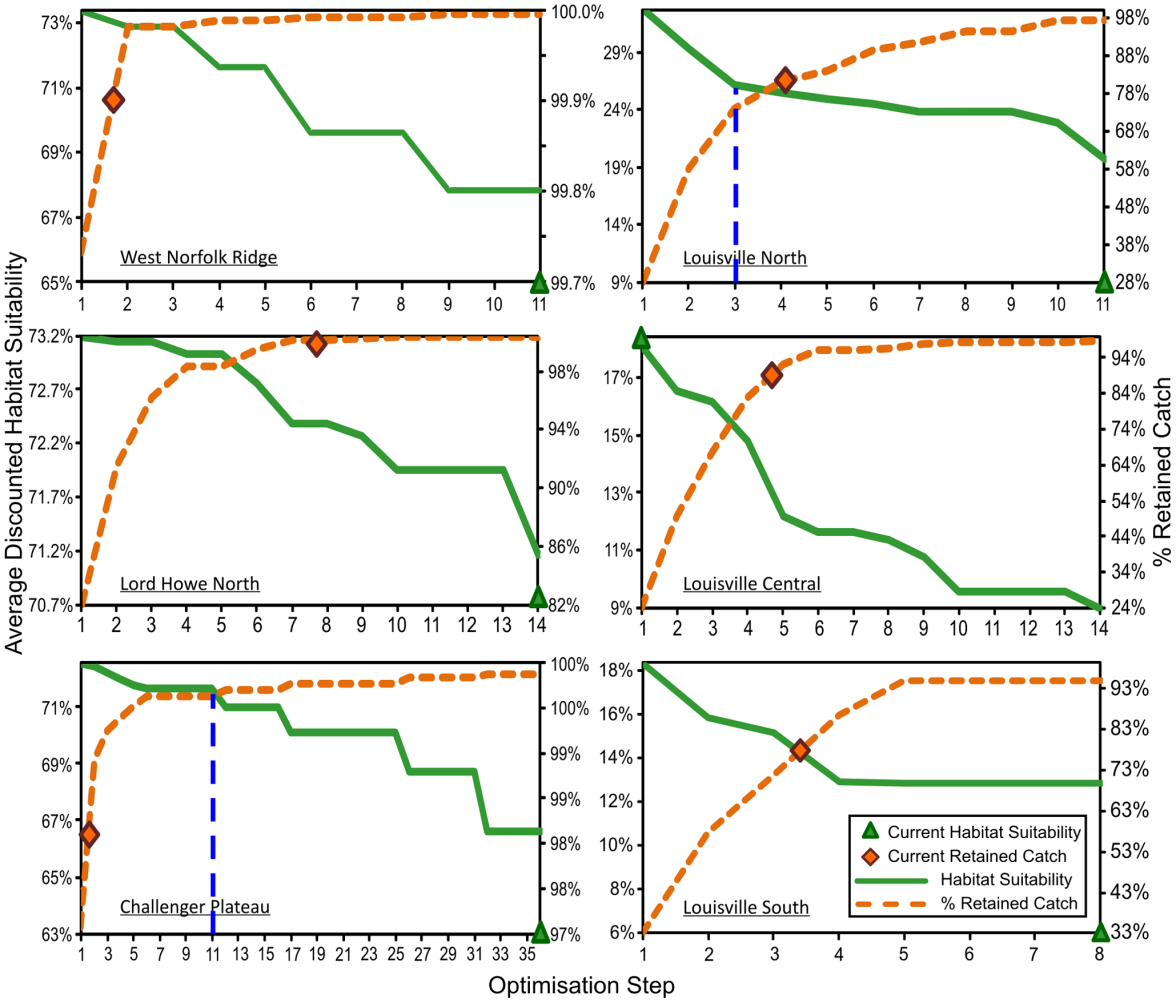
Spatial closure cost-benefit analysis. Cost : benefit curves for six of the New Zealand SPRFMO Area bottom trawl fishing areas, assuming closure of 40% of the blocks in each fishing area, and showing the decline in average, discounted habitat suitability and increase in percentage retained catch (decreasing cost to industry) as blocks of highest historical catch value are sequentially opened. The average discounted habitat suitability and percentage retained catch of current closures in each area (Penney *et al*. 2009) are shown as points on these curves. Blue dashed lines mark the position of the example optimised trade-off closure scenarios chosen for the Challenger Plateau and Northern Louisville Ridge areas, and illustrated in Figure 10.

Cost : benefit trade-off curves for six of the fishing areas are shown in Figure 9. These show the decline in average, all-depths, discounted habitat suitability and the increase in percentage retained catch, moving from closure of 40% of blocks with highest all-depths, discounted habitat suitability, to the closure of 40% of blocks of least cost to industry. The average, all-depths habitat suitability and percentage retained catch of the current spatial closures in each area are shown as points along these trade-off curves. These cost : benefit curves only consider two block categories, closed or open, with no provision for the move-on areas in Penney *et al*. [9], and the current move-on blocks were considered to be open for the purposes of calculating the cost : benefit of current closures.

Comparing the cost : benefit of existing closures (shown by the points on Figure 9) with alternative closure scenarios, it is evident that existing closures have been selected to have low cost in terms of lost catch. This is a direct consequence of the original decision to primarily close lightly fished blocks, and to leave the more heavily fished blocks open [9]. Existing open and move-on areas retain, on average, 88% of historical catch across all fishing areas. In the western region (Challenger Plateau, Lord Howe Rise and West Norfolk Ridge) current closures effectively minimise the costs to industry in terms of lost historical catch, with retained historical catch averaging 93%.

In contrast, even using discounted habitat suitability indices, the average, all-depths habitat suitability of the current closures is below the range that could be achieved under any of the alternative 40% closure scenarios explored for all areas except the central Louisville Ridge (Figure 9). On the Challenger Plateau, the low average habitat suitability of current closures results mainly from the fact that only 15 blocks are currently closed, whereas a 40% closure would require 23 blocks to be closed. Any 23 block closure scenario will increase the average habitat suitability of the closed area on the Challenger Plateau. In the West Norfolk Ridge and Lord Howe North areas, one block more is currently closed than is required by a 40% closure, so the low habitat suitability of current closures does not result from insufficient closures, but results from closure of less suitable blocks.

The cost to industry of current closures along the Louisville Ridge is somewhat higher than for the western region, but retained catch is still well above the chosen 75% optimisation objective, averaging 85% across the eastern region. The predicted habitat suitability of fishable depth areas along the Louisville Ridge is slightly lower than the western region, but is still well above 60%. However, as a result of the small areas of fishable depth in many of the blocks along the Louisville Ridge and the heavy fishing on many of these areas, the discounted all-depths habitat suitability of alternative Louisville Ridge closure scenarios based on entire blocks is low, ranging from 1% to 33%. Fishing effort along the Louisville Ridge has concentrated on those blocks with more fishable depth area. As a direct consequence of closing blocks with lower historical fishing effort, most of which also have small fishable depth areas, the habitat suitability of the current Northern and Southern Louisville closed areas lies well below the range of all alternative closure scenarios. The Central Louisville area is the only fishing area where the average habitat suitability of existing closures slightly exceeds the habitat suitability for alternative closures in that area. This is achieved by closing only four blocks in the higher habitat suitability range for the area (after excluding blocks originally included in error by Penney et al. [9] as a result of erroneous tows), whereas a 40% closure requires the closure of eight blocks.

### Optimisation of Spatial Closures

**Figure 10:**
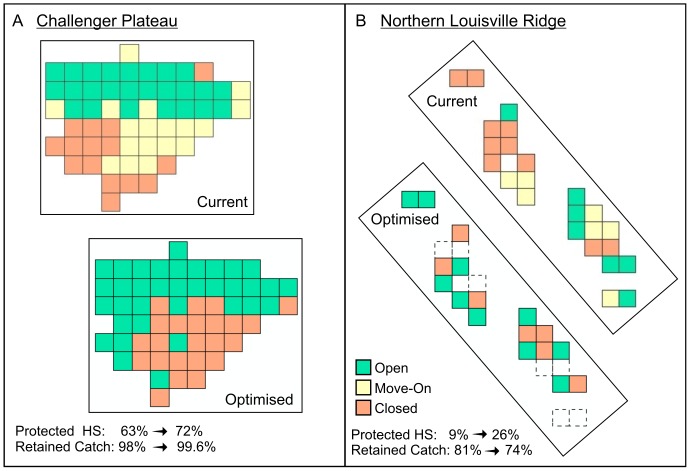
Spatial closure cost-benefit optimisation. Comparison of current and chosen example optimised spatial closures in a) the NW Challenger Plateau and b) the Northern Louisville Ridge fishing areas, assuming closure of 40% of the blocks in each fishing area and objectives of achieving at least 75% of the range in average habitat suitability, and retaining at least 75% of total historical catch, in each fishing area. The optimised scenarios shown use all-depths discounted habitat suitability, and are those marked with the blue dashed lines on the cost : benefit trade-off curves in Figure 9. Dashed line blocks are those deleted from the original trawl footprint after correction of erroneous trawl tow records. The current management approach includes blocks that are open to fishing, but within which a move-on rule is applied (Penney *et al*. 2009). The optimised closures shown do not include a separate move-on management category.

The Challenger Plateau and the Northern Louisville Ridge were chosen to provide two contrasting examples of optimised spatial closures under the chosen management objectives, in areas with different fishing histories and fishable depth areas. The chosen example trade-off positions along the cost-benefit curves for these two areas, which achieve protection of at least 75% of the potential range in average habitat suitability and thereafter minimise costs to industry, are indicated on Figure 9. The block closures corresponding to the chosen optimised scenarios for the two areas were transferred into ArcGIS© and the resulting maps of these ‘optimised’ closure scenarios are compared with the current closures in Figure 10.

There are substantial differences between the effects of optimisation in these two areas. The Challenger Plateau bottom trawl footprint consists of 58 blocks, of which 15 are currently closed, 25 are open and 18 are subject to a move-on rule [9] (Figure 10a, current closures). A 40% closure requires 23 blocks to be closed in this area and the average VME likelihood of the current closures is below the value that could be realised using any 23 block closure (Figure 9). Of the 36 optimisation steps for the NW Challenger Plateau, retained historical catch ranges from 97.1% for scenario 1 to 99.9% for scenario 36, so the objective of retaining 75% of historical catch can be met by any scenario. Average discounted habitat suitability ranges from 73% for scenario 1 to 67% for scenario 36. Scenario 12 achieves 74% of this range in habitat suitability, so scenario 11, which achieves 86% of this range, was chosen as the scenario that minimises cost to industry while still retaining at least 75% of the range in potential habitat suitability. Under this scenario 11 (Figure 10a, optimised closures), the average discounted habitat suitability of closed areas increases from 63% to 72%, while retained historical catch increases from 98% to 99.6%, compared with current closures.

After removal of footprint blocks incorrectly incorporated by Penney *et al*. [9] as a result of erroneous tows (east-west errors), the Northern Louisville Ridge bottom trawl footprint consists of 17 blocks, five of which (29%) are currently closed, six are open and six are subject to a move-on rule [9] (Figure 10b, current closures). A 40% closure requires seven blocks to be closed and the VME likelihood of current closures is below the value that can be achieved by any seven block closure (Figure 9). Of the 11 optimisation steps for the Northern Louisville Ridge area, the retained historical catch ranges from 28% for scenario 1 to 97.3% for scenario 11, and only exceeds 75% from scenario 4 (81%) onwards. Average discounted habitat suitability ranges from 33% for scenario 1 to 20% for scenario 11. Less than 75% of this range is achieved from scenario 2 onwards. There is therefore no scenario that meets both the requirements of retaining 75% of potential habitat suitability range and 75% of retained catch. Scenario 2 achieves 74% of the potential range in protected habitat suitability, so almost achieves the habitat suitability objective. For the purposes of this exercise, scenario 3 was chosen as the trade-off scenario lying between the scenario that achieves the habitat suitability objective (scenario 2) and the one that achieves the retained catch objective (scenario 4). Under scenario 3, (Figure 10b, optimised closures), the average discounted habitat suitability of closed areas increases from 9% to 26%, while the retained catch decreases from 81% to 74%, compared with current closures.

## Discussion

The most fundamental question posed by UN General Assembly Resolutions 61/105 and 64/72 for bottom fisheries on the high seas is: Where are vulnerable marine ecosystems likely to occur? All consequent obligations to protect such ecosystems are dependent on answering that question objectively and reliably. The development of high-resolution benthic habitat prediction models for high-seas areas, such as those of Davies & Guinotte [7] for Scleractinians and Yesson *et al*. [36] for octocorals, provides a cost-effective way of answering this question consistently across large areas, such as the SPRFMO Convention Area.

When combined with quantitative mapping of the distribution of fishing effort, results of predictive habitat models can be used in quantitative assessments of the risk of fisheries interaction with those VMEs, similar to the ‘productivity-susceptibility’ risk assessment plots of Hobday *et al*. [5], [6]. Provided trawl tow-by-tow data are available, measures of seabed area swept can be used to develop discounting factors to quantify the reduction in likelihood of VME occurrence as a result of the impacts of past trawling on swept seabed areas. Either non-discounted or discounted measures of predicted VME habitat suitability can then be used for planning of spatial management measures to protect areas of highest likelihood of VME occurrence, depending on whether the priority is to protect residual VMEs in unfished areas, or to protect and recover areas with the highest predicted coral habitat suitability.

### Cost-benefit Analysis and Evaluation of Current Closures

One of the approaches that has emerged in the planning of high seas spatial protection measures is that of restricting bottom fishing to areas that have already been fished and focussing spatial protection measures on high diversity areas that have not been impacted by fishing. This approach of ‘freezing the footprint’ underlies the SPRFMO bottom fishing interim measures [2], the spatial closures implemented by the North-East Atlantic Fisheries Commission [37] and the ‘open-area’ approach in the U.S. fishery management plans for groundfish in the Bering Sea and Aleutian Islands [38]. This approach also underpins the current New Zealand spatial closures in the SPRFMO Area footprint, where selection of closed, move-on and open areas was based directly on the level of historical (2002–2006) fishing effort in each footprint block [9].

Numerous studies have shown that bottom fishing reduces seabed biodiversity in fished areas, particularly of fragile, habitat forming corals. However, even in heavily fished areas Waller *et al*. [26] found areas of untouched, highly biodiverse seabed on parts of the northwest Atlantic Corner Rise seamounts. Clark & Rowden [24] and Clark et al. [39] report areas of undamaged corals on rough ground on the fished ‘Graveyard’ seamount complex on the Chatham Rise. Some 80% of current deep sea coral and sponge gardens identified using underwater imagery in the Aleutian Islands region are located in areas open to bottom trawling [40], [41]. These coral and sponge gardens have the highest diversity and abundance of deep sea corals and sponges documented in the North Pacific and yet remain open to trawling within the historically fished ‘frozen footprint’.

Unfished areas occurring within ‘fished area’ footprints defined at coarse resolution are likely to contain undamaged benthic communities and may retain high coral likelihood, notwithstanding the fact that bottom fishing has occurred in parts of the footprint. The justification for leaving previously fished areas open to further fishing needs to be based on objective discounting of the biodiversity in swept areas, while recognising the likelihood of remaining biodiversity in un-swept areas. If there is an intention to leave some areas open to fishing while closing others with high likelihood of supporting undamaged VMEs, then use of indices of habitat suitability discounted for the impacts of past fishing can provide some objective justification for focussing spatial protection measures on previously unfished areas, leaving previously fished and substantially impacted areas open to further fishing.

This has important consequences for the planning of spatial closures. Without accounting for the potential effects of past fishing in reducing seabed biodiversity, most blocks in the New Zealand bottom trawl footprint within fishable depths would be of similar coral habitat suitability and any 40% of blocks could be closed to achieve 40% protection of predicted VMEs. In the absence of information on likelihood of VME occurrence at the time the New Zealand bottom fishery impact assessment was prepared, this was the implied logic behind the existing New Zealand closures [9]. These were justified at the time against open and move-on areas using comparisons of seabed topography and depth range, both of which are important determining factors of suitable coral habitat. However, subsequent availability of predictive coral habitat models, quantitative evaluation of seabed swept areas, application of discounting factors for reduced biodiversity in swept areas and estimation of residual coral VME likelihood shows that the existing closures are sub-optimal for protecting likely coral VMEs in all but one of the high-seas fishing areas constituting the New Zealand historical trawl footprint.

### Optimisation of Spatial Closures

The example optimised closure scenarios presented for the Challenger Plateau and Louisville Ridge (Figure 10) illustrate the conservation challenges that result from the different availability of fishable depth in these two areas. On the Challenger Plateau, where large areas of fishable depth exist, the optimised closure example easily meets both the conservation and cost minimisation objectives, resulting in a substantial increase in the average habitat suitability of the protected areas (63% to 72%) while also achieving a slight increase in retained historical catch (98% to 99.6%). All of the existing open blocks would remain open, and the areas open to fishing would be extended to include more of the western slope of the plateau (Figure 10a). The increased benefits would be realised by closing most of the move-on blocks in the southeast-central plateau. It would seem that this win-win alternative should be acceptable to industry and conservation groups.

In contrast, the optimisation objectives cannot be met by any of the explored alternative closure scenarios on the Northern Louisville Ridge. The optimised closure example shown is a compromise that results in a substantial increase in the all-depths habitat suitability of the protected areas (9% to 26%), but this is achieved at the cost of reducing retained historical catch from 81% to 74%. The resulting proposed closures differ substantially from the current closures (Figure 10b), with a number of the currently open blocks being closed and *vice versa*. It seems likely that this alternative closure scenario would be less enthusiastically received, and would require greater discussion with industry and conservation groups. For both of these areas, better consolidation of contiguous open and closed areas may also be preferred, requiring the consultative exploration of further alternative scenarios.

This approach does not address the question of implementation of a move-on rule. Reliance on move-on rules as a primary mitigation measure to avoid significant adverse impacts on VMEs has been increasingly criticised in recent years [42], [43] as being inadequate to protect VMEs, as well as potentially contributing to the spread of fishing effort. Move-on rule weight thresholds have not been supported by studies linking by-catch weights to actual benthic biomass or biodiversity. In many cases, move-on weight thresholds have been set at high levels such that a move-on is seldom triggered [43]. As a result, Auster *et al*. [42] advocate permanent spatial closures as the preferred management response, noting that move-on provisions should only be an initial step towards identification and protection of areas known or likely to contain VMEs.

The optimisation approach taken in this paper is similar to that implemented in the conservation planning software package Marxan [44], which generates optimised closure scenarios based on the spatial distribution of a range of ‘conservation features’, under some specified optimisation objectives. Marxan is generally used to optimise a larger number of features at a finer spatial scale than the approach used here, which was tailored to cost : benefit analysis of the existing closures at 20-minute block resolution using single (albeit integrated) measures of benefit and cost. The approach in this paper therefore falls somewhere between a finer-scale multi-factorial optimisation approach and that adopted during scientific evaluation of alternative spatial closure proposals for the Ross Sea Region [30], where participants proposed alternative closure boundaries and the value and cost of individual alternative proposals were then quantified, but without any explicit optimisation process.

### Options for Improvement and Implementation

In the absence of seabed survey data on benthic community composition and seabed geology, predictive habitat models provide the only source of information with which to objectively evaluate the likelihood of occurrence of VMEs in high-seas areas. However, there is scope for improving these models to reduce shortcomings they have in reliably predicting VME. For example, the global scleractinian habitat model of Davies &Guinotte [7] used here is designed to optimise global habitat suitability predictions based on occurrence data for species which do not necessarily have global distributions. Such models may not optimise habitat predictions for a smaller geographic region where species composition and niche habitat requirements differ, such as the western SPRFMO Area. Deep-sea coral reefs in the northern hemisphere are dominated by *Lophelia pertusa* whereas deep-sea coral reefs around New Zealand are dominated by *Solenosmilia variabilis*. Without true absence data these models tend to over-predict and are not prevented from predicting the occurrence of species that do not actually occur in a region. It should be noted, though, that in doing so these models are predicting a suitable environmental niche for the species included in the model, rather than the presence of the species itself. The niche may well be suited to an alternative species in other regions.

Current global coral habitat suitability models predict high values for scleractinian habitat suitability across the entire depth range of the orange-roughy targeted bottom trawl fishery (Figure 4a), providing low discrimination in predicted habitat suitability across the depth ranges of most interest in fisheries-related risk assessments. Habitat suitability across broad areas with similar depth, such as the Challenger Plateau and Lord Howe Rise areas, will be strongly influenced by seabed geology for which few data are available. Seabed geology or substratum type have therefore not been included in these models, potentially resulting in incorrect prediction of suitable habitat in areas of soft-substratum. Integration of substratum type data into the habitat models would improve the predictions and enable better discrimination of suitable habitat within the fishable depth range. The Davies & Guinotte [7] model also does not include species occurrence data for a number of other habitat-forming taxa included in the New Zealand VME evidence protocol [45], such as Antipatheria (black corals), Alcyonacea (soft corals), Gorgonacea (sea fans) [36], hydrocorals, bryozoans or crinoids, all of which contribute to VMEs in the region. Inclusion of these taxa would make habitat models more useful for identifying areas with a high probability of supporting the full range of key SPRFMO VME taxa. The Maxent modelling software also does not provide estimates of uncertainty in the predicted occurrence of species, without which it is not possible to determine how much confidence one can have in the results. These shortcomings should be addressed if the approaches described in this paper are implemented. High resolution, regionally tailored, predictive habitat models have been developed by Guinotte & Davies [46] for use in assessing deep-sea coral habitat suitability within essential fish habitat area closures and National Marine Sanctuaries in the U.S West Coast Exclusive Economic Zone [46], and by Ross & Howell to predict and map the extent of listed conservation habitats on the United Kingdom and Irish North Sea extended continental shelf [47]. Experience gained in developing those regional models is being applied in a project to develop a regionally optimised predictive habitat suitability model for the western SPRFMO Area, with inclusion of a broader range of VME taxa specific to the region and the application of alternative modelling approaches, including boosted regression trees [A. Rowden, NIWA, New Zealand, pers. comm.].

The most useful improvement that could be made to the reliability of predictive habitat models would be to conduct seabed biodiversity surveys to ground-truth the predictive models. Initial predictive habitat model results can be used to focus survey effort on selected areas where additional biodiversity and presence-absence data would have most power in improving model reliability. New Zealand is intending to conduct such survey work at selected sites in the SPRFMO Area in 2014. The results of ground-truthed, regionally tailored predictive VME habitat prediction models will provide an essential component for spatial planning and management initiatives in the South Pacific region.
